# Supplementary material for: Facial Emotion Recognition Deficit in Children with Moderate/Severe Obstructive Sleep Apnea
Source: Brain Sci. 2022 Dec 8;12(12):1688. doi: 10.3390/brainsci12121688 (PMC9776404; doi:10.3390/brainsci12121688)
Supplement: Supplementary file 1 [file brainsci-12-01688-s001.zip › brainsci-2034920-supplementary.pdf]

Table S1. Correlations between behavioral performance and demographics and PSG characteristics

		**age**	**BMI**	**BMI z-score**	**OAHI**	**TST**	**ArI**	**SpO_2 nadir**	**Emotional symptoms**	**Conduct problems**	**Hyperactivity**	**Peer problems**	**Prosocial behaviors**
RT_{Happy}	r	**-0.669**	**-0.246**	-0.059	-0.027	0.152	0.047	-0.031	-0.002	0.098	0.047	0.012	-0.089
	P	**< 0.001**	**0.004**	0.496	0.756	0.078	0.587	0.716	0.985	0.259	0.590	0.889	0.301
RT_{Sad}	r	**-0.612**	**-0.249**	-0.096	-0.007	0.136	0.040	-0.047	0.057	0.136	0.047	0.012	-0.132
	P	**< 0.001**	**0.003**	0.264	0.938	0.116	0.642	0.591	0.512	0.115	0.587	0.886	0.124
ACC_{Happy}	r	0.141	0.031	0.088	-0.004	-0.080	0.011	0.023	-0.016	-0.106	-0.036	0.037	-0.038
	P	0.102	0.717	0.309	0.966	0.352	0.903	0.792	0.850	0.220	0.676	0.668	0.662
ACC_{Sad}	r	0.067	0.020	0.094	0.013	0.081	-0.002	-0.047	-0.012	**-0.191**	-0.089	-0.001	0.128
	P	0.440	0.816	0.276	0.883	0.348	0.985	0.587	0.887	**0.026**	0.302	0.987	0.137

Significant correlation coefficients (*P* < 0.05) are bolded.

Table S2. Correlations between PSG characteristics and psychosocial and behavioral problems

		**Emotional symptoms**	**Conduct problems**	**Hyperactivity**	**Peer problems**	**Prosocial behaviors**
OAHI	r	**0.188**	0.095	0.068	0.083	-0.094
	P	**0.028**	0.271	0.433	0.338	0.274
TST	r	-0.030	0.053	0.128	-0.010	0.028
	P	0.727	0.538	0.137	0.905	0.747
ArI	r	0.145	0.069	0.020	0.031	-0.128
	P	0.091	0.425	0.819	0.721	0.139
SpO_2 nadir	r	-0.135	-0.125	-0.080	-0.041	0.019
	P	0.118	0.146	0.357	0.635	0.823

Significant correlation coefficients (P < 0.05) are bolded.
